# Assessing the quality of cardiac rehabilitation programs by measuring adherence to the Australian quality indicators

**DOI:** 10.1186/s12913-022-07667-2

**Published:** 2022-02-28

**Authors:** C. M. Astley, A. Beleigoli, R. Tavella, J. Hendriks, C. Gallagher, R. Tirimacco, G. Wilson, T. Barry, R. A. Clark

**Affiliations:** 1 Caring Futures Institute, College of Nursing and Health Science, Flinders University, University Drive, South Australia (SA) 5042 Bedford Park, Australia; 2 Adelaide Medical School, University Adelaide, The Queen Elizabeth Hospital Campus, SA 5011 Woodville South, Australia; 3 Department of Cardiology, Central Adelaide Local Health Network, SA Dept. of Health, SA 5000 Adelaide, Australia; 4 Centre for Heart Rhythm Disorders, University Adelaide and Royal Adelaide Hospital, SA 5000 Adelaide, Australia; 5 Integrated Cardiovascular Clinical Network SA, iCCnet, level 1 Administration Building, 1 Tonsley Boulevard, SA 5042 Tonsley, Australia

**Keywords:** Accreditation, Quality improvement, Cardiac rehabilitation, Acute coronary syndromes, Coronary heart disease

## Abstract

**Background:**

Every year, over 65,000 Australians experience an acute coronary syndrome (ACS) and around one-third occur in people with prior coronary heart disease. Cardiac rehabilitation (CR) aims to prevent a repeat ACS by supporting patients’ return to an active and fulfilling lifestyle. CR programs are efficacious, but audits of clinical practice show variability of program delivery, which may compromise patient outcomes. Core components, quality indicators and accreditation of programs have been introduced internationally to increase program standardisation. With Australian quality indicators (QIs) for cardiac rehabilitation recently introduced, we aimed to conduct a survey in one state of Australia to assess the extent to which programs adhere to the measurement of QIs comparing country, metropolitan, telephone and face to face programs.

**Methods:**

A cross- sectional survey design with face validity testing was used to formulate questions to evaluate cardiac rehabilitation program and personnel characteristics and QI adherence. Between October 2020- December 2021, 23 cardiac rehabilitation programs across country and metropolitan areas were invited to participate. Quality improvement was defined as adherence to the Australian Quality Indicators, and we developed an objective score to calculate program performance categorised by quartiles. Significance of CR completion and time to enrolment between program type (telephone versus face to face) and location (country versus metropolitan were compared using Pearson’s Chi-square and Mann–Whitney U tests.

**Results:**

Among the 23 CR programs, 15 were country and 8 metropolitan-based and 22 were face to face and 1 telephone-based. Median wait time from discharge was 27.0 days, (interquartile range 19.3–46.0) across all programs and country completions of enrolled were 76.9% versus metropolitan 56.5%, *p* < 0.001 and telephone versus face to face 92.9% versus 59.6% *p* < 0.001. Pre-program QI adherence was higher than post program for depression, medication adherence, health-related quality of life and comprehensive re-assessment. Seventy four percent of programs were ranked at a medium level of performance (mean score: 11.4/16, SD ± 0.79).

**Conclusions:**

A survey of 23 cardiac rehabilitation programs, showed variability in adherence to measurement of the Australian Cardiovascular and Rehabilitation Association and Australian Heart Foundation Cardiac Rehabilitation Quality Indicators.

**Trial registration:**

Australia New Zealand Clinical Trials Registry (ANZCTR), ACTRN12621000222842, registered 03/03/2021.

**Supplementary Information:**

The online version contains supplementary material available at 10.1186/s12913-022-07667-2.

## Background

### Scientific background rationale

Every year, over 65,000 Australians experience an acute coronary syndrome (ACS) (heart attack or unstable angina) and around one-third occur in people with prior coronary heart disease (CHD)[1–3]. In Australia CHD has the largest single-disease morbidity (nearly one-fifth of all deaths), at a cost of $1.14 billion annually[4] .Cardiac rehabilitation (CR) aims to prevent a repeat ACS by supporting patients’ return to an active and fulfilling lifestyle [5]. A CR program delivers components of exercise, education and psychosocial assessment support by a skilled multi-disciplinary team [5–7]. Previous studies and systematic reviews have shown efficacy of exercise-based, comprehensive CR to reduce mortality, myocardial infarction, improve functional capacity, psychosocial wellbeing and quality of life in coronary artery disease patients [8–11]. Benefits are also seen in patients with heart failure [12, 13] or those having cardiac surgery [14] and cardiac rehabilitation is also cost effective [15]. Evidence-based CR is therefore able to improve clinical outcomes but practice audits show that comprehensive, exercise-based CR is not being translated into practice and alternative models of delivery are proving effective {5,7,16,17,18] A UK audit of adherence to evidence-based minimum standards in 170 CR programs showed only 30.6% were high performing programs, with 5.3% not meeting any evidence-based minimum standards [6].Two Australian audits have shown variability in CR program duration (3–14 weeks), program length (1–30 sessions), exercise (3–41 sessions), exercise sessions per week {1,2,3,4,5,6,7], exercise duration (15–120 min) and essential education components (68.5–97.6%).[5, 7] Such variability may compromise delivery of evidence-based exercise CR and optimisation of the best outcomes for patients. This is because programs may not produce proven benefits unless translation to practice is done according to the evidence tested in rigorous peer-reviewed studies.[19] This variability may also be exacerbated by many CR programs still using a hospital, out-patient based model of care, established 50 years ago, despite fundamental changes in societal and medical care, and questions whether CR programs meet the needs of patients, as rates of program referral and participation have not improved. [20].

Globally and in Australia, core components [21–24] and quality indicators [25, 26] have been developed to guide implementation of evidenced-based CR program content to help address program variability and drive translation of evidence into clinical practice. Accreditation systems are another tool to drive standardisation of care to deliver evidence-based scope of practice, qualifications, and program content delivery [19]. In the past decade accreditation systems have been implemented in the USA, UK and Europe, but not Australia [19–28].

**Fig. 1:**
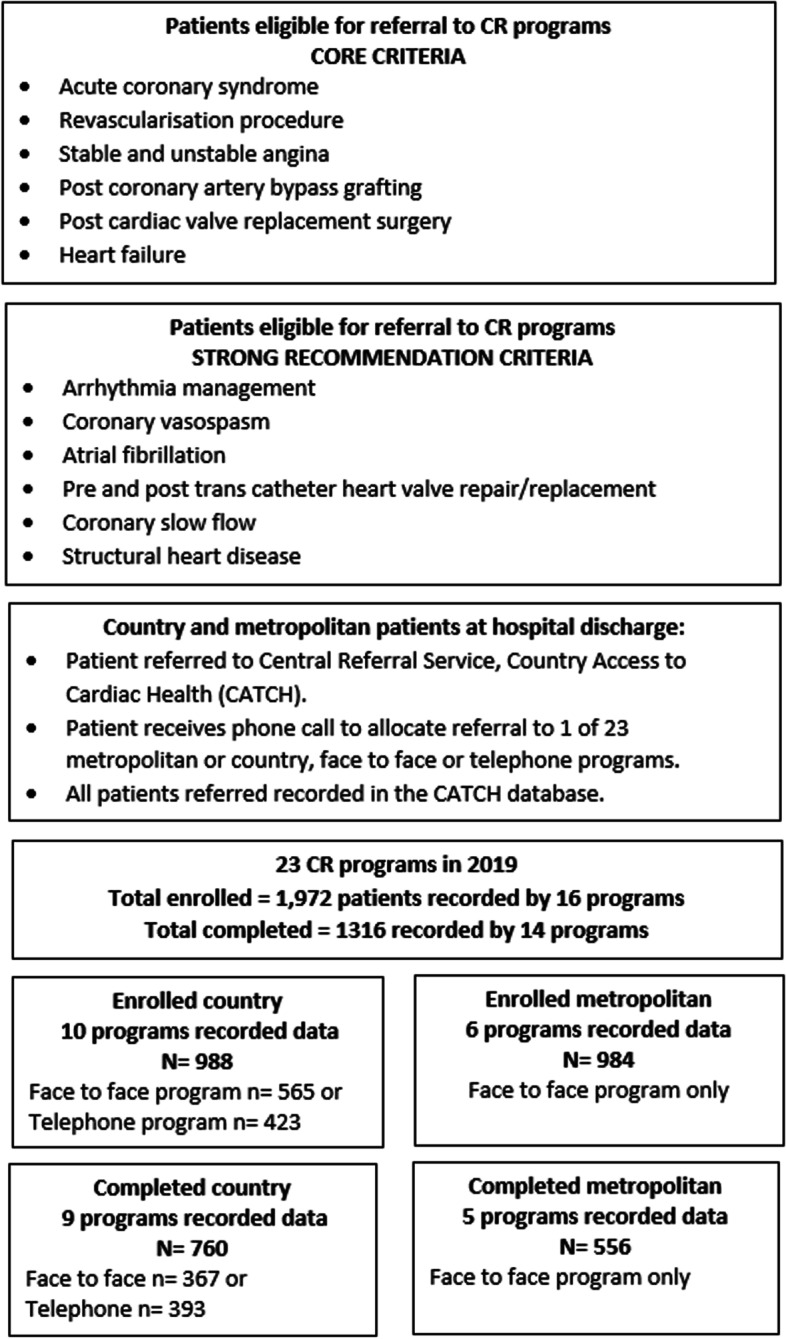
South Australian Cardiac rehabilitations programs

The Commonwealth Government of Australia funds a universal health system (public programs), supported by a private health system, funded by users who make health insurance contributions (private programs) [29]. South Australia is one of 8 Australian states and territories, occupying an area of 983,483 km^2^ (5 times the size of the United Kingdom) with a population of 1.7 million in 2020, with 22.4% living in country areas** [**30]. With equitable access to health services for country patients a challenge in such a geographically large state, a central referral system and telephone cardiac rehabilitation program is available for country and metropolitan patients as well as face to face programs. Patients eligible for cardiac rehabilitation include those with an index admission of acute coronary syndrome, stable angina, revascularisation or valvular procedure/surgery and heart failure (Fig. 1). South Australian cardiac rehabilitation programs record patient level data in the County Access to Cardiac Health database (CATCH), accessible to country and metropolitan programs. An audit of cardiac rehabilitation programs between 2013–2015 revealed that only 30% of those eligible were referred and 30% who were referred attended. Those eligible but not referred (or declined referral) were older, more likely female with an index admission for heart failure or arrhythmia and more comorbidities than those who were referred and attended [16].

**Fig. 2:**
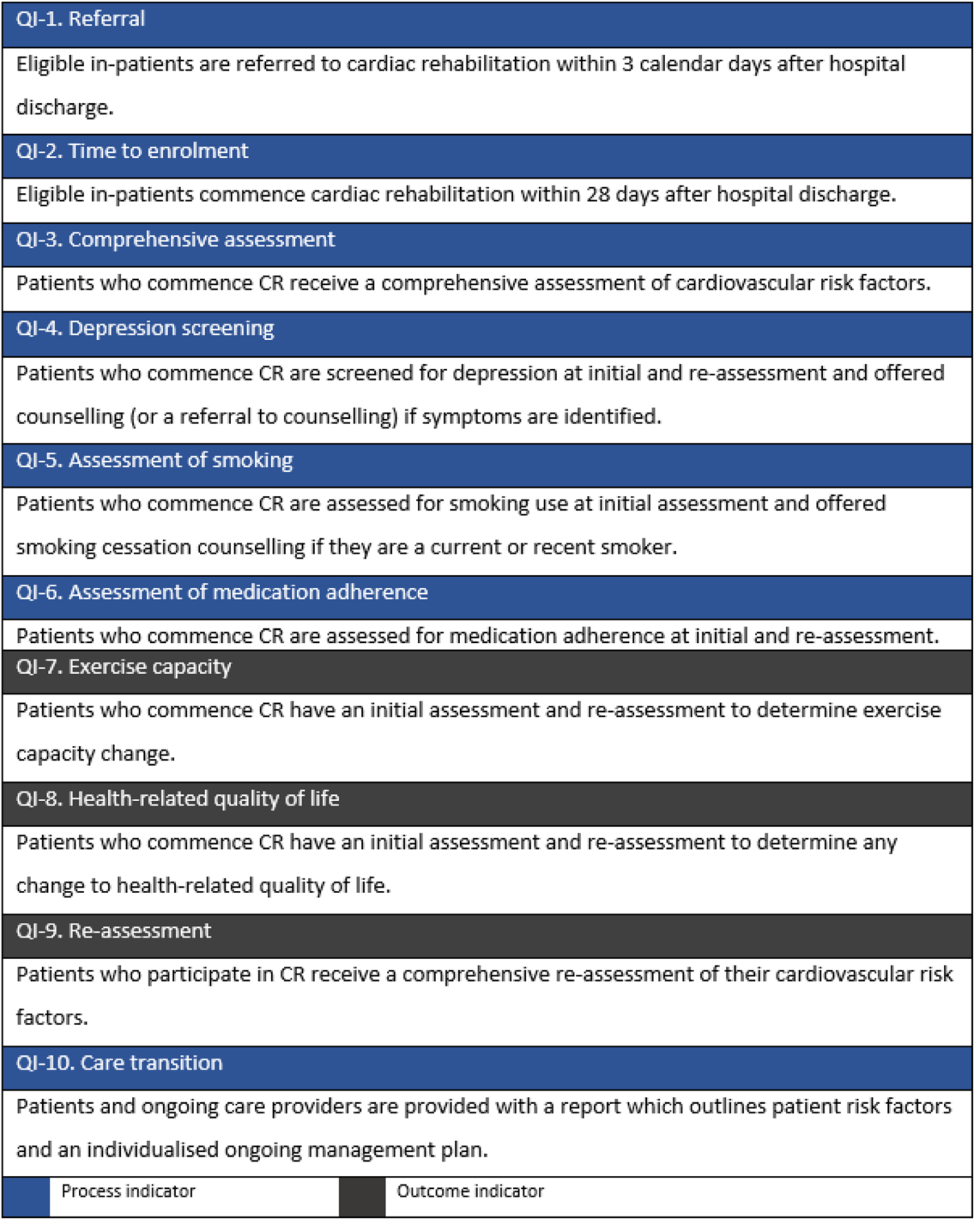
Australian Quality Indicators for cardiac rehabilitation

In 2021 the Australian Cardiovascular and Rehabilitation Association (ACRA) and National Heart Foundation (NHF) published 10 Australian Quality Indicators (QIs) for Cardiac Rehabilitation, (Fig. 2) [31]. As part of a process of change management to facilitate implementation and adoption of an accreditation system, the aim of this study was to assess the extent to which all South Australian (SA) programs adhere to measurement of the national QIs in the content delivery of CR.

## Objectives

1. Describe and compare the characteristics of country and metropolitan, telephone and face to face CR programs and personnel.
2. Measure the performance level of CR programs based upon adherence to the Australian quality indicators.
3. Compare adherence to the national quality indicators between country and metropolitan programs and modes of delivery.

### Why this study is necessary

Quality indicators are one part of the process of standardising and measuring content, personnel and facilities to deliver evidence-based CR programs. Introducing new concepts in clinical practice requires change management to achieve uptake amongst clinicians. This baseline study will raise awareness and contribute to the change management process towards adoption of QI measurement into practice and assist with clinician recognition of the benefits of an accreditation system.

## Methods

### Study design

A cross- sectional survey design was used to formulate questions to evaluate CR program and personnel characteristics and QI adherence. This study is part of the ongoing Country Heart Attack Prevention (CHAP) research program ethical review, Southern Adelaide Clinical Human Research Ethics Committee (HREC) 62.20 with the same HREC deeming the survey as a quality improvement initiative and as such did not require separate ethical review. Informed consent was implied by survey completion and participants were informed of this in the survey introduction, along with a description of the survey purpose and a check box that they understood this process.

### Setting and participants

Between October 2020- December 2021, all 23 public (South Australian Government Department of Health [SA Health]) and private sector cardiac rehabilitation programs who were part of SA Health’s Cardiac Rehabilitation Clinicians Coalition (a workgroup of the Department of Health’s [SA Health]) Cardiology Clinical Network across country and metropolitan areas were invited to participate.

### Variables

#### Survey Development

We built a survey in QUALTRICS™ following review of the ACRA/NHF Core Components and the Australian QIs for Cardiac Rehabilitation [21, 31, 32] and in consultation with CR clinicians from the Cardiac Rehabilitation Clinicians Coalition (*N* = 15). A meeting presented the Australian QIs and discussed the concept of accreditation and how this could also help drive improved program content quality. We then presented the plan for a survey as a way of establishing a baseline of program content adherence with the Australian QIs. All were supportive, with many questions around implementation and process, which we found helpful in our survey development. Face validity testing was conducted on the draft survey including 5 clinicians: 2 country, 2 metropolitan and one from the private sector with the main feedback around how an accreditation program would be managed and reported. The same clinicians were invited to repeat the survey once the final survey was developed and disseminated.

### Survey delivery

Participants were invited and informed through the CR Clinician’s Coalition, (a group representing all program and health services across the state), instructed that the survey should be completed by the primary program coordinator and that only one survey per program was required. As we were delivering the self-reported survey during the COVID19 pandemic, all participants were asked to complete the survey for content, personnel, and program practices for the 2019 time period. Participants were asked to upload evidence to validate some responses such as qualifications, professional education, registration, and comprehensive assessment tools. Accompanying the survey was a Completion Guide with explanatory definitions and instructions.

### Definitions

Quality improvement for this study was defined as adherence to the Australian Quality Indicators (Fig. 2) and variables specific to the CATCH database entry. Program performance was defined as the level of adherence to the QIs for each program. Attendance was defined as attending ≥ 1 session in 2019 and completion was defined as completing ≥ 70% of the program. The completion definition has not been validated but was agreed upon by expert consensus and with ≥ 75% the benchmark used in the European Association for Preventive Cardiology (EAPC) accreditation program [19]. We did not evaluate adherence to QI 1 (Fig. 2) as currently there is not a coordinated data infrastructure in SA to enable programs to measure this.

### Measuring program performance level

To measure and rank the performance level of each CR program we applied a quantitative scoring process related to QI question responses. An independent group of CR content and analytics experts met to develop the scoring criteria. Each QI, its subcomponents and 4 other process questions were given a numerical weighting, with the purpose of placing priority on the quality indicators with the strongest evidence-base. This method was developed by the consensus of a group of experts, (listed in Appendix 1), which was then applied to the responses for each program, to produce a score out of 16.

### Data sources/measurement

Selection bias was minimised by including all public country and metropolitan CR programs in 1 state of Australia. Study size was confined to public and private health system CR programs who are members of the CR Clinicians Coalition.

### Quantitative variables

A survey tool was used to collect data and included 4 sections. A) *Program questions*: related to mode of delivery and length of programs, proportion of education and exercise sessions, participant volumes, utility of evidence-based content guides, data entry into the CATCH database and registration on the Australian Heart Foundations (NHF) Australian online cardiac services directory. B) *Personnel questions*: related to contribution from, and referral pathways to a multidisciplinary team, personnel professional memberships, qualifications, and professional development activities. C) *Quality indicator questions*: related to nine out of ten of the Australian Quality Indicators. D) *Participant experience questions*: asked about the navigability and value of the survey and its outcomes, (Appendix 2).

### Statistical methods

Descriptive statistics are presented as means ± standard deviations (SD) for continuous variables and frequency and percentages for categorical variables. To measure level of performance each program had set criterion applied based upon adherence to the Australian Quality Indicators, aggregated (Appendix 1) and categorised into quartiles: high (13–16), medium (9–12.5), low (5–8.5) and poor (0–4.5). Country and metropolitan programs were compared as were telephone versus face-to-face programs. Percentage of total CR programs meeting and not meeting each of the 9 QIs were calculated. Pearson’s Chi square and Mann–Whitney Utests were used to determine whether completion and median wait time differed between country and metropolitan programs, telephone or face to face reached statistical significance, set at a *p*-value of 0.05 and conducted in SPSS statistical program (version 27).

## Results

Twenty-three cardiac rehabilitation programs were invited and participated in a self-reported survey with a 100% response rate from those invited. Eight programs were from metropolitan locations, (3 from the private health system), 15 programs were from the country, including 1 telephone program.

### Enrolments and completions

Nineteen out of twenty-three (82%) programs reported entering data into the CATCH database. Of 16 (69.5%) programs which could provide enrolment data, there were 1,972 patients who attended ≥ 1 session in 2019. Characteristics of programs in 2019 are presented in Fig. 1.

Country versus metropolitan.

There were 15 country programs and 8 face-to face-programs. Of the 10 (66.6%) country programs providing data, there were 988 enrolments, and of the 6 (75.0%) metropolitans, there were 984 enrolments reported. Of 14 (60.8%) programs that provided data, there were 1,316 patients who completed CR in 2019 reported. Country program patient completions of enrolled were 760/988 (76.9%) and metropolitan were 556/984 (56.5%) and this difference reached statistical significance (*P *< 0.001).

### Telephone versus face to face

There was one telephone program and 22 face-to-face programs. Telephone program enrolments compared to face to face were 423 versus 1549 and telephone enrolments comprised 42.8% of country enrolments in 2019. Comparing telephone completions and face to face there were 393 versus 923 and telephone comprised 51.7% of country completions. Of those enrolled, completions for the telephone program were 393/423 (92.9%) versus 760/1549 (59.6%) for metropolitan and this difference reached statistical significance (*P* < 0.001).

### Waiting time to commence cardiac rehabilitation

Time to enrolment (QI2) from hospital discharge showed a wait time range between 14–57 days. Median wait time was 27.0 days, (interquartile range [IQR] 19.3–46.0) across all programs, with country having a median of 33.0 days (IQR 21–45) and metropolitan a median of 23.0 days (IQR 18–37), *p* = 0.610. Telephone versus face-to-face median wait times were 36.0 (IQR 36–36) versus 24.0 days (IQR 21/45), *p* = 0.800.

Program length and sessions.

Mean total program length was 7.0 weeks (SD ± 1.11) with country slightly longer than metropolitan programs (Table 1). Length of telephone versus face-to-face program was 7.0 versus 6.68 weeks. Sessions per week for telephone versus face-to-face programs were 1.0 versus 1.4. Exercise sessions per week for telephone versus face to face was 0.0 versus 2.17 sessions and education sessions 1.0 versus 1.09 sessions per week.

**Table 1:** Program length and sessions

Program length/sessions	Total, *n *= 23 mean (± SD)	Country *n* = 15 mean (± SD)	Metro *n* = 8 mean (± SD)	Telephone *N *= 1 N	F2F *N* = 22 mean (± SD)
Length of program(mean/wk)	7.0 (1.11)	7.57 (0.75)	6.0 (0.93)	7.0	7.00 (1.42)
Sessions per week(mean/wk)	1.45 (0.50)	1.43 (0.51)	1.50 (0.53)	1.0	1.48 (0.51)
Exercise sessions per week (mean/wk)	1.47 (0.51)	1.46 (0.51)	1.50 (0.53)	0.0	1.47 (0.51)
Education sessions per week (mean/wk)	1.07 (0.31)	0.97 (0.13)	1.25 (0.46)	1.0	1.10 (0.30)

Use of evidence -based content.

All services used an evidence-based standard or framework to guide service content, including the National Heart Foundation (NHF) 2004 CR framework, [33] NHF Pathway to Recovery for CR, (32) or the SA Health CR Model of Care [34] and 19/23 (82%) programs were registered on the National Heart Foundation’s online location directory [35]. The cardiac rehabilitation multidisciplinary team included a nurse and either a physiotherapist or exercise physiologist in all programs. Compared to country, all metropolitan programs had a dietician and pharmacist, but few of all programs had a psychologist (13%), general practitioner (GP) (8.7%) or cardiologist (17.4%) as a regular team member (Table 2). Where there was lack of a specific health professional as a team member, there was a referral pathway available in both groups but slightly less for country programs (Table 3).

**Table 2:** Multidisciplinary professional team member

Profession	Team member, *N* = 23	Country, *n *= 15 n (%)	Metro = 8 n (%)	Telephone *n* = 1 n	F2F *n *= 22 n(%)
Nurse	23	15 (100)	8 (100)	1	22 (100)
Physiotherapist	20	14 (93)	6(75)	0	20 (90)
Exercise physiol	3	1 (6.6)	2(25)	1	2 (9.1)
Dietician	20	12 (80)	8(100)	1	19 (86)
Pharmacist	19	11 (73)	8(100)	1	18 (81)
Social worker	15	10 (66)	5(62)	1	14 (63)
Psychologist	3	1 (6.6)	2(25)	0	3 (13.6)
General practitioner	2	2 (13)	0	0	2 (9.1)
Cardiologist	4	1 (6.6)	3(37)	0	4 (18.2

**Table 3:** Multidisciplinary professional referral pathway

Profession	Referral pathway to, *N* = 23	Country, *n* = 15 n (%)	Metro, *n* = 8 n (%)	Telephone *N* = 1 *n*	F2F *n* = 22 n (%)
Nurse	5	4(26)	2 (25)	0	5 (22.7)
Physiotherapist	11	9(60)	2 (25)	0	11 (50)
Exercise physiology	7	3(20)	4 (50)	0	7(31.8)
Dietician	16	10(66)	6 (75)	0	16 (72.7)
Pharmacist	10	9(60)	1 (12.5)	0	10 (45.4)
Social worker	11	9(60)	2 (25)	0	11 (50)
Psychologist	9	4(26)	5 (62.5)	0	9 (40.9)
General practitioner	15	10(66)	5 (62.5)	0	15 (68.1)
Cardiologist	7	4(26)	3(37)	0	7 (31.8)

### Primary program coordinator characteristics

All the primary coordinators were registered nurses with 78.2% completing a postgraduate qualification relating to cardiovascular care (Masters or Graduate Diploma). Eighty seven percent belonged to the Australian Cardiovascular and Rehabilitation Association (ACRA) peak body. All participated in some type of professional development in 2019 with the majority choosing cardiovascular-based conferences, webinars, workshops, and seminars.

Australian Quality indicator (QI) adherence.

**Fig. 3:**
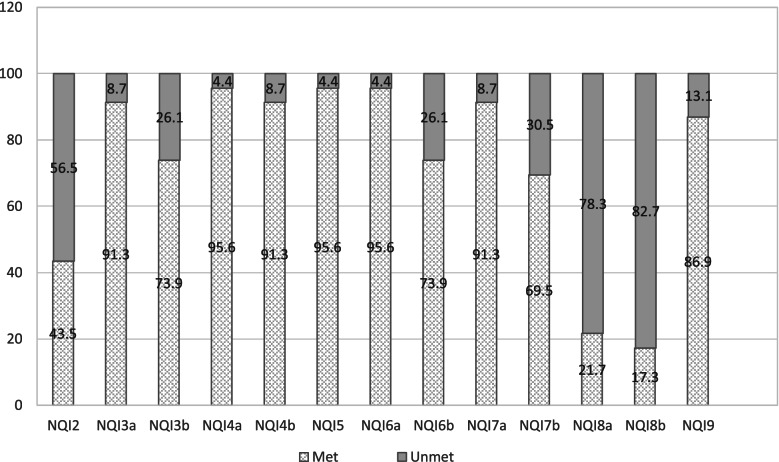
Percentage of Cardiac Rehabilitation Programs Meeting and Not meeting the Quality Indicators

All QIs except QI 1 were measured by some programs and program adherence is presented in Table 4. Pre-program adherence was higher than post program for depression, medication adherence, health-related quality of life and comprehensive re-assessment. The telephone program did not measure exercise capacity (QI 7) and health- related quality of life (QI 8) was poorly measured pre and post program (21.7% versus 17.3%) Fig. 3. For depression screening (QI 4) 11(47.8%) of programs used both the Patients Health Questionnaire (PHQ) 9 and 2, with 7 (30%) using the PHQ9 only. For functional exercise capacity assessment (QI 7), the six-minute walk test was used by 16 (69.5%) of programs. Participant experience showed that 19 (82.6%) found the survey very easy or easy to navigate and 21 (91.3%) strongly agreed or agreed that there is value in developing an accreditation system for CR programs in South Australia.

**Table 4:** Australian Quality indicator adherence (31)

Quality indicator (QI)	Total *N* = 23 n (%)	Country *N* = 15 n (%)	Metropolitan *N *= 8 n (%)	Telephone *N* = 1 y/n	Face to face *N* = 22 n (%)
QI 2.0	10 (43.5)	6 (40.0)	4(50)	y	9(40.9)
QI 3.0	21 (91.3)	14 (93.3)	7(87.5)	y	20(90.9)
QI 4.0	22 (95.6)	15 (100)	7(87.5)	y	21(95.4)
4.1	21 (91.3)	14 (93.3)	7(87.5)	y	20(90.9)
4.2	21 (91.3)	14(93.3)	7(87.5)	y	20(90.9)
QI 5.0	23 (100)	15(100)	8(100)	y	22(100)
5.1	21(95.6)	14(93.3)	7(87.5)	y	20(90.9)
5.2	22 (95.6)	15(100)	7(87.5)	y	21(95.4)
QI 6.0	22(95.6)	14(93.3)	8(100)	y	21(95.4)
6.1	17 (73.9)	12(80)	5(62.5)	n	17(77.3)
QI 7.0	21 (91.3)	13(86.6)	8(100)	n	21(95.4)
7.1	21 (91.3)	13(86.6)	8(100)	n	21(95.4)
QI 8.0	5 (21.7)	4(26.6)	1(12.5)	n	5(22.7)
8.1	4 (17.3)	3(20)	1(12.5)	n	4(18.2)
QI 9.0	17 (73.9)	11(73.3)	6(75)	n	17(77.3)
QI 10.0	20 (82.6)	13(86.6)	7(87.5)	y	19(86.3)

*QI-2*: Time to enrolment, *QI-3*: Comprehensive assessment, *QI-4*:Depression screening, 4.1:Refferal to counselling, 4.2: Depression re-assessment, *QI-5*: Assessment of smoking, 5.1 Referral to counselling, 5.2: Smoking re-assessment, *QI-6*: Assessment of medication adherence, 6.1: Medication adherence re-assessment, *QI-7*: Assessment of exercise capacity, 7.1 Exercise capacity re-assessment, *QI-8*: Assessment of health-related quality of life(HrOL), 8.1; HrQOL re-assessment, *QI-9*: Comprehensive re-assessment, *QI-10*: Care Transition

*QI* = Quality indicator; *QIa* = pre-program; *QIb* = post program -assessment

*QI-2*: Time to enrolment, *QI-3a&b*: Comprehensive assessment, *QI-4a&b*: Depression screening, *QI-5*: Assessment of smoking, *QI-6a&b*: Assessment of medication adherence, *QI-7a&b*: Assessment of exercise capacity, *QI-8a&b*: Assessment of health-related quality of life, *QI-9*: Care Transition

### Program Performance

Seventy four percent of programs were ranked at a medium level of performance with a mean performance score of 11.4/16, (SD ± 0.79). No programs were ranked as poor, 13% ranked low and 13% high (Table 5). Comparisons between country and metropolitan showed similar results as did telephone versus face to face, where both rated in the medium performance category, (10.7/16) (versus a face to face mean of 11.4 [± SD 1.87]) (Table 5).

**Table 5:** Cardiac rehabilitation Program Performance

Program performance category	Total *N* = 23 n (%)	Country *n* = 15 n (%)	Metro *n* = 8 n (%)	Telephone *n *= 1	Face to face *n* = 22
Poor (0–4.5)	0	0	0	0	0
Low (5–8.5)	3 (13.04)	2 (13.3)	1(12.5)	0	3 (13.6)
Medium (9–12.5)	17 (73.9)	11 (73.3)	6 (75.0)	10.7	16 (72.7)
High (13–16)	3 (13.04)	2 (13.3)	1(12.5)	0	3 (13.6)

## Discussion

The aim of this study was to assess the extent to which programs met the national QIs. This study surveyed 23 cardiac rehabilitation services in South Australia, Australia for program and personnel characteristics and quality performance adherence, comparing country and metropolitan, telephone and face to face programs. In 2019, of the 69.5% of programs that could provide data, there were 1,972 patients enrolled with 66.7% of these patients completing. Country and metropolitan enrolments were similar with 42.8% of country patients attending the telephone program. Country completions were higher than metropolitan (76.7% v 56.5%, *p* < 0.001) and the telephone program had higher completions than face to face programs (92.9% v 59.6%, *p* < 0.001). We were unable to measure total patients eligible in this dataset and this is something that our quality improvement initiatives hope to address in the future. However, an audit conducted between 2013- 2015 amongst the same programs showed that approximately 16,600 patients per year are eligible and of these 5,000 are referred. [16].

### Program and personnel characteristics

Mean total program length was 7.0 weeks (± SD 1.11) across country and metropolitan, telephone and face to face. Program length and sessions were similar amongst country and metropolitan, telephone and face to face programs (mean 7.0 weeks or 10.5 sessions) and indeed 6–8 weeks (12–16 sessions) is the norm across Australian programs.(5, 7) However, if we look at international benchmarks, program duration is far less in Australia than our international counterparts in the UK and Europe, who have a standard of ≥ 24 -36 sessions or around 12 -18 weeks, raising the question of what ‘dose’ of CR is most effective to improve outcomes? [19, 36] Total median wait time was 27.0 days (IQR 19.3–46.0), where Australian QI 2 recommends 28 days from hospital discharge (Fig. 2).(31) While metropolitan programs were within the limit of this benchmark (23.0 days, IQR 18–37), country programs, including telephone, were a longer wait time (median 33.0 days IQR 21–45 and 36 days IQR 36–36, respectively). Internationally, the recommended wait time from referral to start of CR is 14–28 days [19].

The ideal cardiac rehabilitation program consists of a multidisciplinary team. In South Australia where programs do not have all types of professionals as part of their team, this is enabled by referral pathways. The multidisciplinary team amongst country, metropolitan, telephone and face to face programs consisted of a nurse and either a physiotherapist or exercise physiologist and to a slightly lesser degree a dietician or pharmacist. Where they were not part of the team, they were accessible via a referral pathway, with the exception of the telephone program (Table 3), which could be an area for improvement, as well as increased access to social workers and psychologists overall.

### Quality indicator adherence

The most measured QI was comprehensive assessment across country, metropolitan, telephone and face to face programs, followed by depression screening, smoking assessment and counselling thereof (Table 5). There is room for program improvement however, as health-related quality of life was the least measured and most programs (73.9%) scored a medium level of performance across country and metropolitan, telephone and face to face services in health-related quality of life, exercise, and comprehensive re-assessment (Table 4). Comparing South Australian performance scores with 3 other combined Australian jurisdictions, representing 39 programs (New South Wales, Australian Capital Territory and Tasmania) showed higher levels of performance than South Australia (High- Aust: 18% versus SA: 13%, Medium/moderate—Aust. 76.9% versus SA: 73.9% and Low-Aust.: 5.1% versus SA: 13%) (Table 5) [37]. Comparing South Australian (SA) program performance scores with the United Kingdom (UK) (6), showed more programs in the middle/medium category (SA: 73.9% versus UK: 45.9%) and SA showing zero poor performance programs compared to the UK’s 5.3%, but the UK showing more programs in the high performance category (UK: 30.6% versus SA: 13.0%) (Table 4). Building exercise capacity measurement into the telephone program could also be done by using a 13-item self-report measure called the Specific Activity Questionnaire (SAQ) [38]. The scoring method of the tool can be used to estimate Metabolic Equivalent of Task (METS) and the questionnaire is publicly available, free of charge and has been validated against exercise stress testing in cardiac patients [38, 39]. Exercise advice could be delivered through mobile health applications or websites in combination with telephone support [40].

### Are the quality indicators and performance score an accurate measure of quality?

A key finding of this survey was the higher completion rates in country compared to metropolitan, and telephone compared to face to face programs. This was likely driven by 42.8% of patients accessing country programs attending the telephone option. While this measure reflects a process indicator, we also know from the survey that the telephone program delivers only one exercise session, where efficacy evidence tells us that exercise is a significant driver of improved CR outcomes [8, 11, 12, 14, 23]. More rigorous data in the form of objective validation is therefore required to confirm the self-reported survey responses and associations with clinical outcomes.

Quality improvement measurement and comparisons often require a multi-component approach of standardisation, measurement, reporting and change management. We were not able to conduct a validation process because of the variability of data entry into the CATCH database. This identifies the need for universal data entry to accurately measure program quality and will require a change management strategy to achieve. Accreditation can be a tool to promote change management among clinicians to drive standardisation of care and deliver evidence-based scope of practice, qualifications, and program content delivery [19].

### Limitations

This survey was self-reported for each program and thus is subject to reporting bias. We were not able to objectively validate the questionnaire responses nor correlate these with clinical outcomes, thus we don’t know whether QI adherence is associated with better quality program content. Further, as the performance score is derived from program adherence to the QIs, the discriminatory capacity of the score may be limited. Since data entry into the CATCH database is not mandatory across the 23 programs, not all survey items measured have 23 programs as the denominator. Further enrolment and completion numbers are underestimated. We were not able to measure enrolment against eligible or referred patients for 2019, though we know from our previous audit work that approximately 5,000 patients are referred to cardiac rehabilitation each year in SA. (16) The sample size of 23 programs is small to measure any meaningful differences between country and metropolitan, telephone and face to face programs. Further the Australian Quality Indicators were only published in 2020 and therefore programs have not had time to implement improvements.(31) Despite this we have surveyed 23 CR programs across SA, evaluating program and personnel characteristics and determining adherence with the Australian Quality indicators, giving us an indication of the level of program quality to inform development of an accreditation system.

## Conclusion

A survey of 23 cardiac rehabilitation programs in one state of Australia, showed variability in adherence to measurement of the Australian Cardiovascular and Rehabilitation Association and National Heart Foundation Cardiac Rehabilitation Quality Indicators as measured by a survey. These data indicate that there are gaps in the delivery of evidence-based content in South Australian cardiac rehabilitation programs, which could be addressed by quality improvement initiatives such as an accreditation system. A state-wide engagement and education program will be required as an essential preliminary step towards an accreditation program.

## Supplementary Information

**Additional file 1.** Cardiac Rehabilitation Program Performance Scoring Criteria.

**Additional file 2.** The South Australian Cardiac Rehabilitation Program Accreditation Survey.

## Data Availability

The dataset used and/or analysed during the current study are available from the corresponding author on reasonable request.

## Abbreviations

ACS: Acute coronary syndromes
CHD: Coronary heart disease
CR: Cardiac rehabilitation
ACRA: Australian Cardiovascular and Rehabilitation Association
NHF: National Heart Foundation
QI: Australian Quality Indicators
SA: South Australia
CHAP: Country heart attack prevention research program
HREC: Human research ethics committee
SA Health: South Australian Government’s Department of Health
COVID19: Coronavirus
SD: Standard deviation
CATCH: Country Access to Cardiac Health
GP: General practitioner
PHQ: Patient health questionnaire
SAQ: Specific Activity questionnaire
METS: Metabolic Equivalent of Task
NHMRC: National Health and Medical Research Council
