# Correction to: Assessing the quality of cardiac rehabilitation programs by measuring adherence to the Australian quality indicators

**DOI:** 10.1186/s12913-022-07726-8

**Published:** 2022-03-16

**Authors:** C. M. Astley, A. Beleigoli, R. Tavella, J. Hendriks, C. Gallagher, R. Tirimacco, G. Wilson, T. Barry, R. A. Clark

**Affiliations:** 1 Caring Futures Institute, College of Nursing and Health Science, Flinders University, University Drive, South Australia (SA), Bedford Park, 5042 Australia; 2 Adelaide Medical School, University Adelaide, The Queen Elizabeth Hospital Campus, Woodville South, SA 5011 Australia; 3 Department of Cardiology, Central Adelaide Local Health Network, SA Dept. of Health, Adelaide, SA 5000 Australia; 4 Centre for Heart Rhythm Disorders, University Adelaide and Royal Adelaide Hospital, Adelaide, SA 5000 Australia; 5 Integrated Cardiovascular Clinical Network SA, iCCnet, level 1 Administration Building, 1 Tonsley Boulevard, Tonsley, SA 5042 Australia

**Correction to: BMC Health Serv Res 22, 267 (2022)**

**https://doi.org/10.1186/s12913-022-07667-2**

Following publication of the original article [1], the authors identified two errors in the author names. Firstly, the two co-authors’ names J Hendriks and C Gallagher were mistakenly merged into one name ‘Hendriks Gallagher JC’. Secondly, the given name and family name were erroneously transposed.

The author group has been updated above and the original article [1] has been corrected.
