# Supplementary material for: Assessing the quality of cardiac rehabilitation programs by measuring adherence to the Australian quality indicators
Source: BMC Health Serv Res. 2022 Feb 28;22:267. doi: 10.1186/s12913-022-07667-2 (PMC8883249; doi:10.1186/s12913-022-07667-2)
Supplement: Supplementary file 1 — Additional file 1. Cardiac Rehabilitation Program Performance Scoring Criteria. [file 12913_2022_7667_MOESM1_ESM.docx]

**Appendix 1. Cardiac Rehabilitation Program Performance Scoring Criteria**

| Quality indicator | | Indicator components | Scoring | | Total | |
| --- | --- | --- | --- | --- | --- | --- |
| Do you enter data into the state CR database for every patient who commences the program? | | No/yes | 0.5 | | 0.5 | |
| Is your program registered the NHF CR services directory? | | No/Yes | 0.5 | | 0.5 | |
| Is your program based upon a CR framework, standard or guideline tool to inform program content? | | No/Yes | 0.25 | | 0.5 | |
|  |  | Indicate tool | 0.25 | |  |  |
| QI 2. What was the average number of days that CR participants waited to commence your program from discharge? | | Number required | 1.5 | | 1.5 | |
| QI 3. Do patients who commence CR receive a comprehensive assessment of cardiovascular risk? | | No/yes | 0.5 | | 1.0 | |
|  |  | Upload assessment tool | 0.5 | |  |  |
| QI 4.0 Do patients who commence CR receive screening for depression at initial visit? | | 1. No/yes | 1 out of 4 scores 0.25 | | 1.5 | |
|  |  | 1. Screening tool | 2 out of 4 scores 0.8 | |  |  |
| QI 4.1. If positive for depression are patients offered counselling (or referral to counselling)? | |  | 3 out of 4 scores 1.2. | |  |  |
| QI 4.2. Do patients who commence CR receive an assessment of depression at the re-assessment visit? | |  | 4 out of 4 scores 1.5 | |  |  |
| QI 5.0 Do patients who commence CR receive an assessment of smoking status at the initial visit? | | No/yes | 1 out of 3 scores 0.5 | | 1.5 | |
| QI 5.1 Do you offer smoking cessation counselling (or referral to counselling) if your patients is a current or recent smoker? | |  | 2 out of 3 scores 1.2 | |  |  |
| QI 5.2 Do patients who commence CR receive an assessment of smoking status at the re-assessment visit? | |  | 3 out of 3 scores 1.5 | |  |  |
| QI 6.0 Do patients who commence CR receive an assessment for medication adherence at the initial visit? | 1. No/yes | | | 1 out of 3 scores 0.5 | | 1.5 |
|  | 1. How do you measure? | | | 2 out of 3 scores 1.2 | |  |
| QI 6.1 Do patients who commence CR receive an assessment for medication adherence at the re-assessment visit? |  | | | 3 out of 3 scores 1.5 | |  |
| QI 7.0 Do patients who commence CR have an assessment of exercise capacity at the initial visit? | 1. No/yes | | | 1 out of 3 scores 0.5 | | 2.0 |
|  | 1. How do you measure? | | | 2 out of 3 scores 1.5 | |  |
| QI 7.1 Do Patients who commence CR have a re-assessment of exercise capacity at the re-assessment visit? |  | | | 3 out of 3 scores 2.0 | |  |
| QI 8.0 Do patients who commence CR receive an assessment of health-related quality of life? | 1. No/yes | | | 0.5 | | 1.5 |
|  | 1. How do you measure? | | | 0.5 | |  |
| QI 8.1 Do patients who commence CR receive and assessment of HrQOL at re-assessment visit? |  | | | 1 | |  |
| QI 9.0 Do patients who participate in CR receive a comprehensive re-assessment of cardiovascular risk factors? | No/yes | | |  | | 1 |
| QI 10.0 Do patients and their GP receive a report which outlines patient risk factors and an individualised ongoing management plan? | No/yes | | |  | | 1.5 |
| What was the percentage of patients that completed (Definition ≥ 70% attendance) your program in 2019? | Number required | | |  | | 1 |
| TOTAL |  | | |  | | 16 |
