# Supplementary material for: Assessing the quality of cardiac rehabilitation programs by measuring adherence to the Australian quality indicators
Source: BMC Health Serv Res. 2022 Feb 28;22:267. doi: 10.1186/s12913-022-07667-2 (PMC8883249; doi:10.1186/s12913-022-07667-2)
Supplement: Supplementary file 2 — Additional file 2. The South Australian Cardiac Rehabilitation Program Accreditation Survey. [file 12913_2022_7667_MOESM2_ESM.docx]

**Appendix 2. The South Australian Cardiac Rehabilitation Program Accreditation Survey**

Cardiac Rehabilitation Program Accreditation survey:

This is the baseline survey for the South Australian Cardiac Rehabilitation Credentialing Pilot project. This project was deemed by the Southern Adelaide Human Research Ethics Committee as a quality improvement activity that does require ethical review. Therefore, by completing this survey you are consenting to participate. 
 
As the primary coordinator of your program you are invited to participate. Where there are lead coordinators who job share, we encourage you to fill out one survey for your program together. Where you are involved in more than one program, please complete a survey for each program.
 
We acknowledge that the COVID19 period has affected the operation of CR programs, so we ask that you complete this survey for your program’s **usual content** but please feel free to indicate all the modes you are delivering CR through**.** 
  
Please complete all the fields by referring to the **Survey Completion Guide** attached to the email containing the link to this survey. The system will allow you to leave an incomplete survey and return without losing data.

 You will be asked to upload or provide evidence showing that your program meets the requirements for credentialing (program documentation). If you upload the incorrect document just re-upload with the correct one and the system will replace old with new.
 
If you have any questions, please contact XXX

PROGRAM QUESTIONS

Q1 What is the name, address, email, phone contacts of the primary coordinator(s) of your cardiac rehabilitation (CR) or secondary prevention (SP) program?

- Name and address of program (4) __________________________________________
- Primary Coordinator(s) (7) ________________________________________________
- Contact email address (6) ________________________________________________
- Reception contact (if applicable) (9) __________________________________
- Local health network (if applicable) (10) _________________________________
- Please indicate the funding model for your program ie: permanent on-going / annual review / integrated / medicare chronic disease plan / other? (11) ____________________________
- Do you have a gymnasium facility or similar attached to your program location? (yes/no/or describe) (12) ________________________________________________

Q2 Please indicate the modes of delivery of your program (select all that currently apply):

- Face to face (1)
- Telephone (2)
- Videoconferencing (3)
- Web-based (4)
- Text messaging (5)
- GP practice-based (6)
- Other (7) ________________________________________________

Q3 Please indicate the total length of your program in weeks, number of sessions per week and number of exercise and education sessions per week:

- Number of weeks (1) _________________________________________
- Number of sessions per week (2) _________________________________
- Number of exercise sessions per week (3) __________________________
- Number of education sessions per week (4) _________________________

Q4 What was the total number of patients who enrolled in your program and attended at least one session, in 2019? Enter ND (not data) if you do not know.

Q5 Do you enter data into the Country Access to Cardiac Health (CATCH) database for every patient who commences the program?

- No (1)
- Yes (2)

Q6 Is the your program registered on the Heart Foundation Cardiac Rehabilitation Services Directory?
You can check by copying and pasting this link into your URL:
https://www.heartfoundation.org.au/cardiac-services-directory

- No (1)
- Yes (2)

Q7 Is the content or structure of your program based upon a cardiac rehabilitation framework, standard or guideline?

- No (1)
- Yes, please record which one (2) _________________________________

Q8 Which health professionals do you have working **regularly** within your service? Select all that currently apply:

- Nurse (1)
- Physiotherapist (2)
- Psychologist (3)
- Exercise Physiologist (4)
- Social worker (5)
- Dietician (6)
- Pharmacist (7)
- Cardiologist (8)
- General Practitioner (9)
- Other, please provide (10) ________________________________________________

Q9 Which health professionals do you **refer out** to in your service? Select all that currently apply:

- Nurse (1)
- Physiotherapist (2)
- Psychologist (3)
- Exercise Physiologist (4)
- Social worker (5)
- Dietician (6)
- Pharmacist (7)
- Cardiologist (8)
- General Practitioner (9)
- Other, please provide (10) ________________________________________________

PERSONNEL QUESTIONS

Q10
Are you a Registered Practitioner with the Australian Health Practitioners Association (AHPRA)?

- No (2)
- Yes, please provide your registration number. Enter ND (no data) if you do not know. (1) ________________________________________________

Q11 Are you a member of a relevant professional organisation involved in the delivery of cardiac rehabilitation? (select all that apply)

- Australian Cardiovascular & Rehabilitation Association (ACRA) (1)
- Exercise & Sports Science Australia (ESSA) (2)
- Cardiac Society of Australia and New Zealand (CSANZ) (3)
- European Society of Cardiology (ESC) (4)
- ESC Association of Nursing & Allied Health Professionals (ACNAP) (5)
- American College Of Cardiology (ACC) (6)
- American Heart Association (AHA) (7)
- Other, please list (8) ________________________________________________
- None (9)

Q12 As primary CR/SP coordinator for your program, what is your professional role?

Professional role (1)

▼ Registered Nurse (1) ... General Practitioner (9)

Q13
Have you completed any postgraduate qualifications relating to cardiovascular care?

- No (2)
- Yes, please provide name of course, institution and year of completion. Enter ND (no data) if you do not know. (3) ________________________________________________

Q14 In the **last 12 months** which of the following professional development activities did you complete? (Select all that apply).

- Attend a cardiology conference (1)
- Attend a post graduate cardiac course (2)
- Attend ACRA webinars (3)
- Attend Heart Foundation workshops/seminars/webinars (5)
- Attend other cardiac-specific updates, please list (4) ____________________
- None (13)

QUALITY INDICATOR QUESTIONS

Q15 What was the average number of days that CR referral participants waited to commence CR from discharge in 2019? Enter ND (no data) if you do not know. _______________________________

Q16 DEPRESSION SCREENING
Do patients who commence CR receive screening for depression at initial visit?

- No (2)
- Yes (3), please provide the name of your current depression screening questionnaire/tool (5) _______

Q17 DEPRESSION SCREENING Do patients who commence CR receive **re-assessment** of depression screening to determine a change?

No (2)

- Yes (3)

Q18 DEPRESSION
If positive for depression, do you offer counselling (or referral to counselling)?

- No (2)
- Yes (3)

Q19 COMPREHENSIVE ASSESSMENT
 Do patients who commence CR receive a comprehensive assessment of cardiovascular risk factors? Please upload evidence below.

- No (4)
- Yes, please upload a copy of your assessment tool below (6)

Q19 Comprehensive assessment - upload here:

Q20 RE-ASSESSMENT
 Do patients who participate in CR receive a comprehensive **re-assessment** of their cardiovascular risk factors to determine the change?

- No (2)
- Yes (1)

Q21 ASSESSMENT OF SMOKING
Do patients who commence cardiac rehabilitation have an assessment of smoking status at the initial visit?

- No (2)
- Yes (1)

Q22 ASSESSMENT OF SMOKING
Do patients who commence cardiac rehabilitation have a **re-assessment** of smoking status to determine the change?

- No (1)
- Yes (2)

Q23
SMOKING Do you offer smoking cessation counselling (or referral to counselling) if your patient is a current or recent smoker?

- No (1)
- Yes (2)

Q24 ASSESSMENT OF MEDICATION ADHERENCE
Do patients who commence CR receive an assessment of medications at the initial visit?

- No (2)
- Yes, how do you measure medication adherence? (3) _________________________________

Q25 ASSESSMENT OF MEDICATION ADHERENCE
Do patients who commence CR receive a **re- assessment** of medications to determine adherence?

- No (1)
- Yes (2)

Q26 EXERCISE CAPACITY
Do patients who commence CR have an assessment of exercise capacity at the initial visit?

- No (1)
- Yes, how do you measure exercise capacity? (4) ___________________________________

Q27 EXERCISE CAPACITY
 Do patients who commence CR have a **re-assessment** of exercise capacity to determine a change?

- No (1)
- Yes (2)

Q28 HEALTH-RELATED QUALITY OF LIFE (HRQoL)
 Do patients who commence CR receive an assessment of HRQoL at the initial visit?

- No (2)
- Yes, how do you measure HRQoL? (3) ___________________________________________

Q29 HEALTH-RELATED QUALITY OF LIFE (HRQoL)
 Do patients who commence CR receive a **re-assessment** of HRQoL to determine a change?

- No (1)
- Yes (2)

Q30 In the CATCH database, completion of CR is defined as 70% or greater.
Do you record the number of patients who complete your program?

- No (4)
- Yes, please record how many patients in total completed 70% or more of your program in 2019? (5) _____________________

Q31 CARE TRANSITION
Do patients and their GP receive a report which outlines patient risk factors and an individualised ongoing management plan?

- No (2)
- Yes (3)

YOUR EXPERIENCE

Q32 Overall and with the supporting resources, I found navigating this survey:

- Very easy (1)
- Easy (2)
- Difficult (3)
- Very difficult (4)
- Comment if required (5) ________________________________________________

Q33 I can see the value in undertaking a credentialing process

- Strongly agree (1)
- Agree (2)
- Disagree (3)
- Strongly disagree (4)
- Comment if required (5) ________________________________________________

Q76 We are interested in your thoughts on further development of the accreditation program, please enter any comments below:

________________________________________________________________

Thankyou for your participation, we appreciate your time and input.
